# 

**Published:** 1899-04

**Authors:** 


### BUFFALO MEDICAL JOURNAL

Established 1845 by AUSTIN FLINT, M. D.

EDITORS:

THOS. LOTHROP, M. D. WM. WARREN POTTER, M. D.

ASSOCIATE EDITORS:

WILLIAM C. KRAUSS, M. D.

JOHN A. MILLER, PH. D.

ERNEST WENDE, M. D.

JAMES WRIGHT PUTNAM, M. D.

JOHN PARMENTER, M. D.

ALVIN A. HUBBELL, M. D.

LIBRARY
SURGEON GENERAL'S OFFICE
APR.-1--1899

SCRIPTA FERUNT ANNOS

EUROPEAN AGENCY, J. B. BAILLIÈRE & FILS, 19 Rue Hautefeuille, Paris.
*Subscription, if paid in advance, $2.00; otherwise, $2.50. Foreign subscription, $2.50.*
Copyright 1899, by WILLIAM WARREN POTTER, M. D.
Entered at the Post Office at Buffalo, N. Y., as second-class mail matter.

A. T. BROWN PRINTING HOUSE, BUFFALO, N. Y.

Table of Contents on Page V.

